# Persistent Fever Despite Antitubercular Therapy: Unmasking Haemophagocytic Lymphohistiocytosis Triggered by Disseminated Tuberculosis

**DOI:** 10.7759/cureus.98451

**Published:** 2025-12-04

**Authors:** Alison Galea, Nicholas Tabone Adami, Thelma Xerri, Daniel Farrugia, Chantal Vella

**Affiliations:** 1 Internal Medicine, Mater Dei Hospital, Msida, MLT; 2 Infectious Disease, Mater Dei Hospital, Msida, MLT; 3 Hematology, Mater Dei Hospital, Msida, MLT

**Keywords:** cytopenia, disseminated tuberculosis, fever, haemophagocytic lymphohistiocytosis, intravenous corticosteroids

## Abstract

Haemophagocytic lymphohistiocytosis (HLH) is a rare, life-threatening condition that can complicate various infections, including *Mycobacterium tuberculosis* infection. We describe the diagnostic challenges encountered in a 37-year-old male from Nepal, who presented with dyspnoea and fever. Cross-sectional imaging showed disseminated tuberculosis (TB) with extrapulmonary manifestations. Persistent high-grade fever despite antitubercular treatment and empirical antibiotics, along with generalised lymphadenopathy and trilinear cytopenia, triggered further invasive investigations. Bone marrow aspirate showed haemophagocytosis, confirming HLH secondary to disseminated TB. The patient showed marked clinical improvement when intravenous corticosteroids were administered with antitubercular treatment. This case illustrates an uncommon presentation of a relatively common condition, and the importance of early consideration of HLH, especially in those with persistent unexplained high-grade fever and cytopenia.

## Introduction

Haemophagocytic lymphohistiocytosis (HLH) is an under-recognised, life-threatening, hyperinflammatory syndrome characterised by excessive immune activation and cytokine release [1], with an estimated annual incidence of 1.2 cases per million individuals [2]. Despite its rarity, HLH is associated with a high mortality rate, ranging from 50% to 70%. If not treated, the median survival time is less than two months [3]. The main features, such as persistent fever, hepatosplenomegaly, cytopenia, liver dysfunction, hypertriglyceridemia, and marked hyperferritinaemia, are non-specific and most often overlap with sepsis, septic shock, and other inflammatory disorders, leading to diagnostic delays [1]. 

HLH is classified as primary (genetic) or secondary, related to infections, malignancies, and autoimmune conditions. Among infection-associated HLH, Epstein-Barr virus (EBV) remains the most common culprit [1]. Tuberculosis (TB) has been implicated in 9-25% of infection-associated cases, with survival rates ranging from 40% to 60% [4]. A more recent systematic review on TB-associated HLH estimated an overall mortality rate of 39% [5]. Recognising HLH in the context of TB and HLH is particularly challenging as both present with prolonged fever, cytopenias, and systemic inflammation. These overlapping features lead to diagnostic delays and contribute to the increased mortality rate. Initiation of both anti-tuberculous therapy and immunomodulation is vital [4].

We report a case of disseminated TB complicated by secondary HLH, to highlight the diagnostic and therapeutic challenges and to emphasise that persistent fever despite antitubercular therapy should prompt consideration of HLH and early immunomodulation to prevent fatal outcomes.

## Case presentation

**Figure 1:**
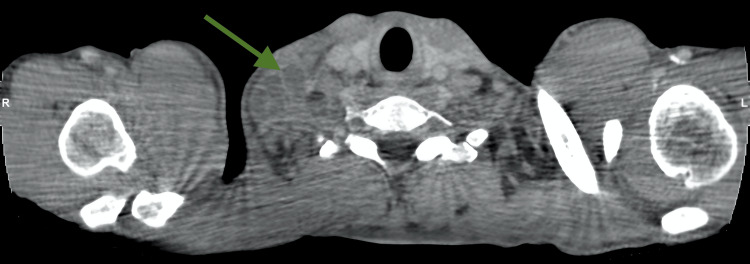
CT Neck and Trunk, axial view. Green arrow indicates enlarged right supraclavicular lymph node with necrotic centres

A 37-year-old Nepalese male cleaner, with no significant past medical history, presented in July 2024 with a haemorrhagic shock secondary to an upper gastrointestinal bleed. Cross-sectional computed tomography (CT) shown in Figure 1 revealed multiple enlarged right supraclavicular lymph nodes with necrotic centres, raising suspicion of lymph node TB. Lymph node biopsy was scheduled to be done electively, given that no active pulmonary involvement was identified. The patient, however, failed to attend subsequent follow-up appointments following discharge. 

**Figure 2:**
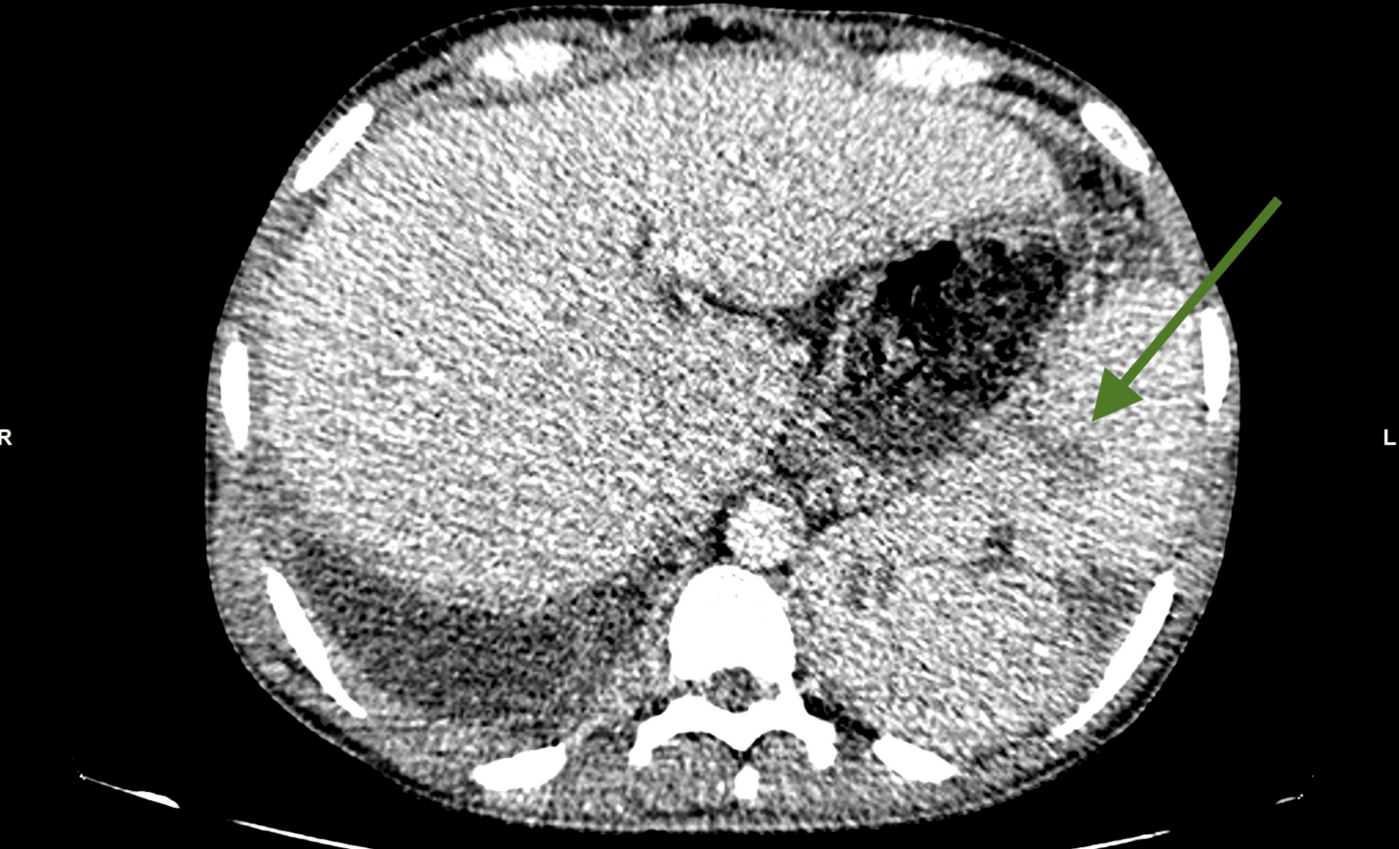
CT Neck and Trunk, axial view, showing hepatosplenomegaly. Green arrow shows splenic nodules

He re-presented nine months later with exertional dyspnoea, fever, and malaise. Repeat CT Neck and Trunk was suggestive of disseminated TB with pulmonary nodules, mediastinal, para-aortic, necrotic supraclavicular, and right axillary lymphadenopathy with right upper and lower lobe consolidation, splenic nodules, and ascites (Figure 2). 

The patient was scheduled for an ultrasound-guided biopsy of the necrotic right supraclavicular lymph node. On presentation, he appeared cachectic (BMI 17.5kg/m^2^), jaundiced, and febrile (38.9°C). He gave a two-day history of high-grade fever and productive cough. On examination, there was a palpable right supraclavicular lymph node, hepatomegaly (four finger breadths below the costal margin), and splenomegaly (two finger breadths below the costal margin). Cardiovascular and neurological examinations were unremarkable. Blood pressure was recorded to be 95/60 mmHg, heart rate 125 beats per minute, and oxygen saturation of 97% on room air. 

**Figure 3:**
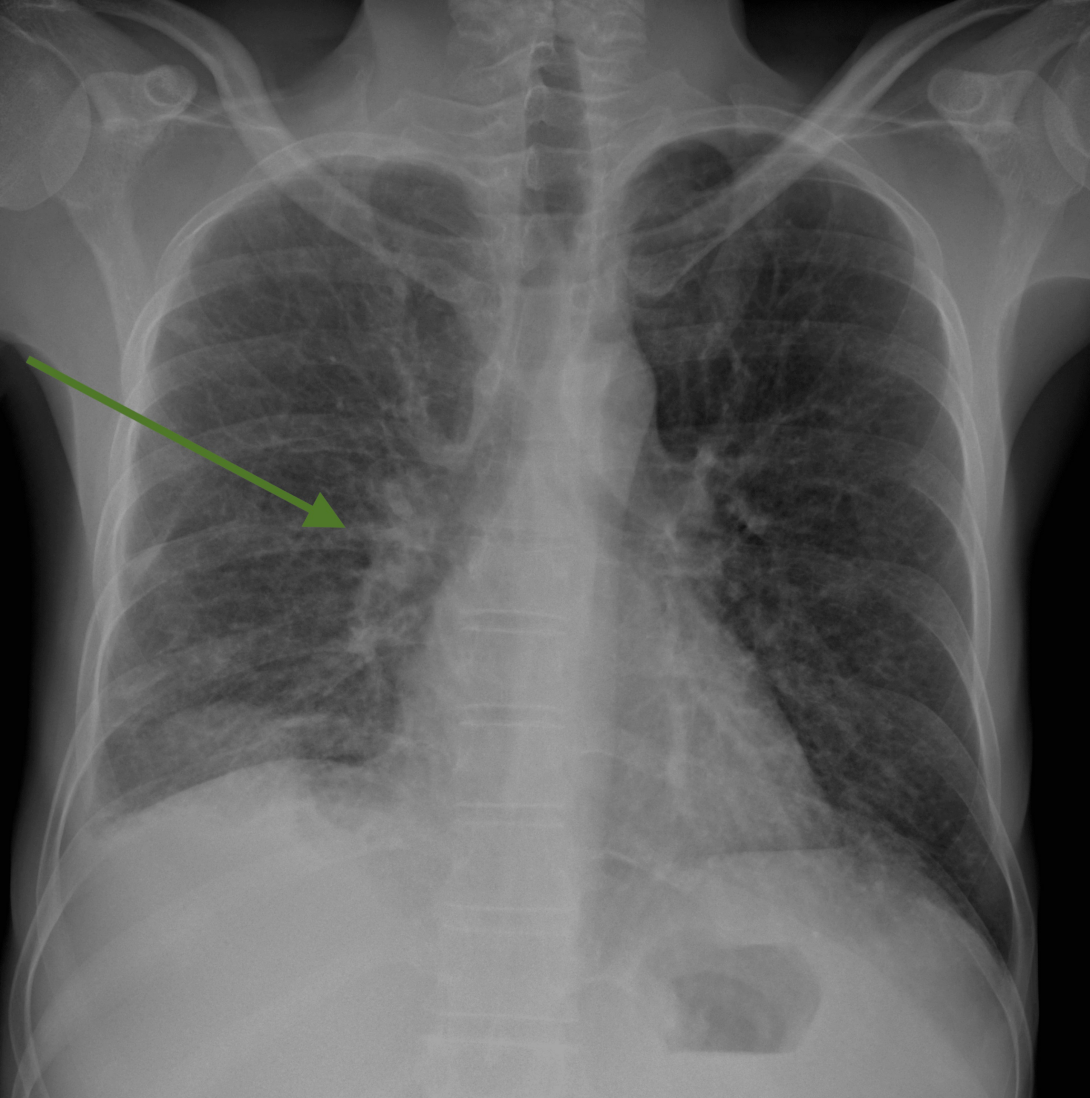
Chest X-ray showing mediastinal lymphadenopathy (green arrow), with pulmonary nodules and right-sided pleural effusion

**Figure 4:**
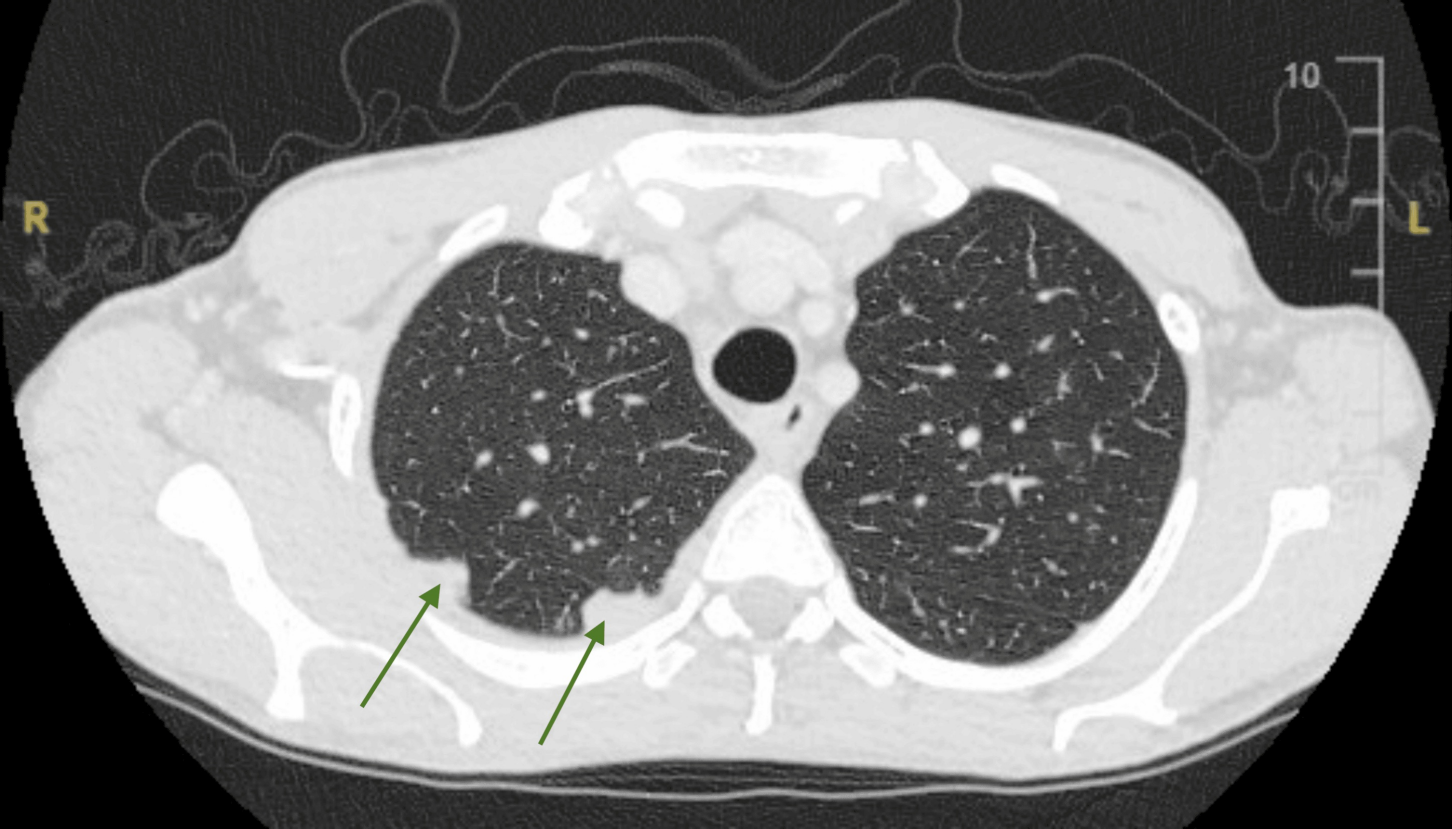
CT Thorax (lung window), axial view, showing two pleura-attached nodule opacities in the right apex posteriorly (arrows)

Initial blood investigations revealed pancytopenia (hemoglobin 9.7g/dL, WCC 1.48x10^9^/L, and platelets 49x10^9^/L), hyponatremia (119 mmol/L), and cholestatic liver dysfunction. Both C-reactive protein (CRP) and erythrocyte sedimentation rate (ESR) were raised (96.8 mg/L and 29 mm/hour, respectively). Chest X-ray revealed a small right-sided pleural effusion with pulmonary nodules and mediastinal lymphadenopathy (Figure 3). CT thorax shows solid pleura-attached nodules (Figure 4). Table 1 shows blood investigations on admission and on day 21. 

**Table 1:** Laboratory findings on admission (day 0) and on day 21 '-' refers to blood test not available ESR: erythrocyte sedimentation rate; CRP: C-reactive protein; ALP: alkaline phosphatase; GGT: gamma-glutamyl transferase; ALT: alanine transaminase; eGFR: estimated glomerular filtration rate

Blood Investigations	Day 0 Results	Day 21 Results	Normal Range
Haemoglobin	9.7	7.8	14.1-17.2g/dL
Neutrophils	0.93	1.94	2.10-7.20 x109/L
Platelets	49	102	146-302 x109/L
Triglycerides	3.16	-	0.1-1.7 mmol/l
Ferritin	2097	-	22-322ng/mL
ESR	38	58	2-10mm 1st Hr
CRP	134.8	57	0-5mg/L
Bilirubin	63.7	28.1	0-21umol/L
ALP	229	216	40-129U/l
GGT	105	173	8-61 U/l
ALT	60	38	5-41U/l
Sodium	119	131	135-145mmol/l
Potassium	4.07	4.13	3.5-5.1mmol/l
Creatinine	48	40	49-104umol/l
eGFR	181	223	mls/min/1.73m^2 ^

Microbiological work-up, including blood, sputum, and urine cultures, was negative. Viral, autoimmune, and parasitic screens (including HIV, EBV, cytomegalovirus, parvovirus, hepatitis panel, malaria, and leishmania) were unremarkable. SARS-CoV-2 polymerase chain reaction (PCR) and respiratory screen were negative. The Ziehl-Neelsen stain was negative. Mycobacterial blood cultures were repeated twice, both yielding a negative result. Sputum examination of mycobacteria was not available, as the patient was unable to provide a sputum specimen. 

Ophthalmic review excluded any possible ophthalmic involvement of TB. No vegetations or TB involvement were seen on transthoracic echocardiography. Histology from ultrasound-guided biopsy of the right supraclavicular lymph node revealed necrotising granulomatous lymphadenitis, suspicious for an infectious aetiology. 

Antitubercular treatment with Rifinah, pyrazinamide, ethambutol, and pyridoxine was started empirically. Given persistent high-grade fevers up to 39.8°C, broad-spectrum antibiotics (piperacillin-tazobactam, ceftriaxone, metronidazole, and amikacin) were administered sequentially, but with no improvement. 

**Figure 5:**
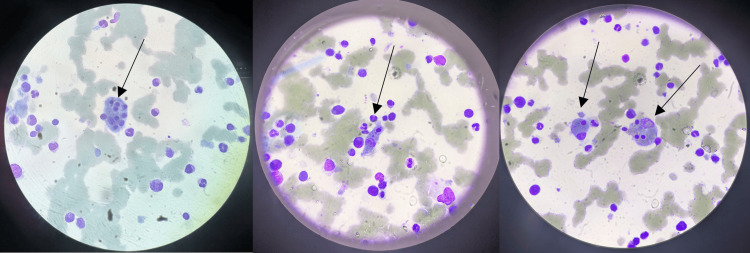
Bone marrow aspirate smear demonstrating hemophagocytosis. Several macrophages (indicated by arrows) contain engulfed hematopoietic cells, including nucleated red cell precursors and leukocytes, consistent with hemophagocytic activity

**Figure 6:**
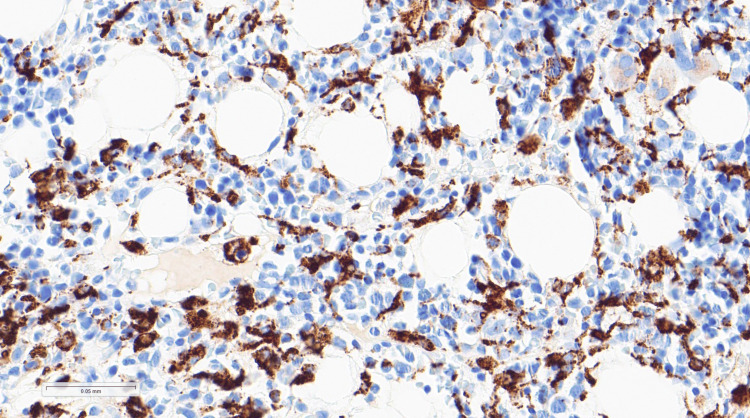
Immunostaining highlights an increased number of macrophages and monocytes with a diffuse interstitial distribution. The cytoplasmic positivity surrounds and occasionally engulfs normal haematopoietic elements, consistent with haemophagocytosis (CD68, ×40)

Intravenous hydrocortisone was started on day 13 of admission, due to suspicion of immune reconstitution syndrome, with a good response. However, the fever recurred on administration of oral prednisolone. Given the persistent fever and pancytopenia as demonstrated in Table 1, bone marrow aspirate was performed on day 21 of admission, showing haemophagocytosis as shown in Figure 5. Figure 6 shows CD68 by immunohistochemistry on a trephine. Notably, the H score prior to bone marrow examination was 200, corresponding to an estimated 80-88% probability of haemophagocytic syndrome. Following confirmation of haemophagocytosis on bone marrow aspirate, the score increased to 235, indicating a 98-99% probability of haemophagocytic syndrome. Subsequent TB PCR (liquid culture) was positive, confirming disseminated TB with no resistance to rifampicin and isoniazid. 

An alternative explanation of fever was suspected when only intravenous corticosteroids were shown to be effective. Once a diagnosis of HLH secondary to disseminated TB was established, hydrocortisone was carefully converted to oral prednisolone after seven days. After 36 days of hospitalisation, the patient was discharged on antitubercular therapy and a tapering course of corticosteroids, with close follow-up. Adjunctive immunomodulatory therapies such as intravenous immunoglobulins (IVIG) were not indicated in this case, given clinical and biochemical improvement on corticosteroids.

## Discussion

According to the 2024 WHO Global Tuberculosis Report, TB is the world’s leading infectious disease killer, surpassing COVID-19 in 2023 [7]. Although TB is typically confined to the lungs, extrapulmonary TB can occur. The lymph nodes, in particular thoracic lymph nodes, are one of the most common sites in extrapulmonary disease [8]. Lymph nodes may act as niches where TB can persist, disseminate, and reactivate [8]. Delayed or inadequate treatment of TB may complicate matters further, leading to multi-organ dysfunction. 

In the current case, cross-sectional imaging performed a year earlier had already revealed incidental findings suggestive of lymph node TB, providing an early clue to the underlying diagnosis. Persistent fever, cytopenia, and hepatosplenomegaly despite antitubercular therapy raised suspicion for lymphoma or another infiltrative disorder. Sarcoidosis was considered but deemed less likely given the necrotising granulomas on lymph node biopsy. Confirmation of *Mycobacterium tuberculosis* by liquid culture required six weeks, delaying diagnostic certainty. Ultimately, the diagnosis of disseminated TB with secondary HLH was established through bone marrow trephine biopsy. 

HLH is a severe hyperinflammatory syndrome characterized by increased pro-inflammatory cytokines, excessive macrophage activation, and phagocytosis of mature blood elements [9]. HLH is classified into primary (or familial), caused by genetic defects in lymphocyte cytolytic activity, usually presenting in childhood, and secondary (acquired), triggered by infections, malignancy, or autoimmune conditions [1].

A systematic review by Koumadoraki et al. identified two studies that reviewed 71 patients with HLH secondary to *M. tuberculosis* infection [1]. Similar to our case, most often, patients were noted to present with fever and hepatosplenomegaly. 

Given that both TB and HLH have overlapping, non-specific symptoms, precise diagnostic criteria are difficult to establish. In the setting of disseminated TB, if clinical deterioration is noted despite antitubercular treatment, especially with underlying cytopenias and coagulation disorders, co-existing HLH should be considered [5]. 

Diagnostic criteria for HLH

The HLH 2024 criteria describe the diagnostic criteria for HLH. These are based on HLH 2004 criteria, excluding natural killer (NK)-cell activity. Fulfilling five or more of the HLH 2024 criteria is highly diagnostic for HLH [10]. Our patient satisfied six out of the seven criteria for HLH: persistent high-grade fever, splenomegaly, hyperferritinemia, hypertriglyceridemia, trilineage cytopenia, and haemophagocytosis on bone marrow biopsy. The level of soluble CD25 (sCD25) was not assessed due to limited local availability. Nonetheless, this case reinforces that in resource-limited settings where sCD25 or NK-cell assays are unavailable, HLH diagnosis can still be confidently established using the modified HLH-2004 criteria.

The H Score is a validated scoring system designed to help clinicians diagnose secondary HLH in adults, comprising nine clinical, biologic, and cytologic variables, appropriately weighted [6]. The H score in our patient was 235, with a diagnostic probability of 98-99% in terms of HLH diagnosis. Table 2 shows the different values of the H score with respect to the case described.

**Table 2:** H Score calculations Reference: Hôpital Saint-Antoine AP-HP [11] AST: aspartate aminotransferase

Parameters		Score	Case Score
Fever	<38.4	0	-
	38.4-39.4	33	-
	>39.4	49	49
Organomegaly	Absent	0	-
	Hepatomegaly or Splenomegaly	23	-
	Hepatomegaly and Splenomegaly	38	38
Cytopenia	Single Cell Line	0	-
	Two Cell Lines	24	-
	Three Cell Lines	34	34
Triglycerides (mmol/l)	<1.5	0	-
	1.5-4	44	44
	>4	64	-
Fibrinogen (mg/dL)	>250	0	0
	<250	30	-
AST (IU/L)	<30	0	0
	>30	19	-
Ferritin (ng/L)	<2000	0	-
	2000-6000	35	35
	>6000	50	-
Haemophagocytosis	Absent	0	-
	Present		
Immunosuppression	Absent	0	0
	Present	18	-
			Total Score: 235

For stable patients, treatment of secondary HLH requires identification and treatment of the underlying cause. For unstable patients, corticosteroids, with or without IVIG, are recommended as first-line treatment [5]. 

Appropriate management of TB-associated HLH is crucial. In 116 patients with TB-associated HLH reviewed by Fauchald et al., the overall survival rate was 55%. There was a 66% survival rate when both antitubercular therapy and immunomodulation were administered, compared to 56% in those who only received antitubercular therapy. Absence of antitubercular therapy resulted in fatal outcomes [4]. 

The diagnostic delays in the case described were multifactorial. Initially, suspicion of TB arose incidentally following cross-sectional imaging performed while the patient was in an intensive care setting. Findings were most consistent with lymph node TB, with no radiological evidence of active pulmonary involvement. Further complicating the diagnostic process was the difficulty in re-establishing contact with the patient after discharge, which delayed lymph node biopsy and likely postponed definitive diagnosis and treatment. This delay may have contributed to the subsequent development of disseminated TB complicated by secondary HLH. 

The initial microbiological investigations, including blood cultures and a complete septic screen, were negative for *M. tuberculosis*. Ultimately, *M. tuberculosis* was isolated on liquid culture six weeks after admission, by which time the diagnosis of HLH had already been established through bone marrow trephine, confirming TB as the precipitating trigger. Hyponatremia noted on admission was hypothesised to result from TB-related adrenal involvement; however, further endocrinological evaluation could not be performed because of the patient’s clinical deterioration and the need to initiate corticosteroid therapy.

Although several cases of TB-associated HLH have been reported, most describe courses requiring immunosuppressive therapy, especially IVIG [5]. The patient’s favourable response to corticosteroids without IVIG or etoposide highlights the variability in disease severity and the potential for recovery when early immunomodulation is initiated. 

## Conclusions

This case highlights the diagnostic challenges of secondary HLH precipitated by disseminated TB and the importance of early consideration of HLH in patients with persistent unexplained fever and cytopenia despite adequate antimicrobial treatment. Our patient’s favourable response to intravenous corticosteroids without the need of additional immunosuppressive therapy, emphasises the spectrum of clinical severity. Early recognition and prompt immunomodulatory treatment of both HLH and TB are crucial to prevent fatal outcomes. 
